# Life of Sir William Tennant Gairdner, K.C.B., M.D., LL.D., F.R.S., Regius Professor of Medicine in the University of Glasgow

**Published:** 1913-03

**Authors:** 


**Life of Sir William Tennant Gairdner, K.C.B., M.D., LL.D., F.R.S., Regius Professor of Medicine in the University of Glasgow.** By GEORGE ALEXANDER GIBSON, M.D., D.Sc. Pp. ix, 817. Glasgow: James Maclehose & Sons. 1912. Price 10s. 6d. net.

An additional and melancholy interest attaches to this biography of the author's old friend, which will be of great interest to the generation of students who were associated with him in his clinical work, as the accomplished author has now himself passed from the scene of his active labours. Sir W. Gairdner was born in 1824, and his death occurred suddenly in 1907, in his 84th year. The affliction from which he suffered was the Adams-Stokes syndrome, consisting in bradycardia, due to complete heart block, attended by attacks of unconsciousness and sometimes slight convulsions. He took the deepest interest in the development of his own symptoms, discussed them freely, and wrote an account of the course of his illness in 1902. The autobiographical sketch attracted great attention when published by Dr. W. T. Ritchie and Dr. Gibson.[1] The volume concludes with a large selection of papers on general and medical subjects which were contributed by the author to various journals, a fragment of the literary work which emanated from his fertile brain.

[1] *Edinb. M. J.*, 1909 (New Series), ii. 315, 507.

